# Conformable thin-film cuff electrodes as tools for peripheral nerve recording: a comparative study

**DOI:** 10.3389/fnins.2026.1930438

**Published:** 2026-09-09

**Authors:** Amparo Güemes, Margaux O. A. Forner, Ariadna Guindo-Arroyo, Ruben Ruiz-Mateos Serrano, George G. Malliaras, Alejandro Carnicer-Lombarte

**Affiliations:** 1 Department of Engineering, Institute for Biomedical Innovation, University of Cambridge, Cambridge, United Kingdom; 2 Electrical Engineering Division, Department of Engineering, University of Cambridge, Cambridge, United Kingdom; 3 Department of Biomedical Engineering, City University of Hong Kong, Kowloon, Hong Kong SAR, China

**Keywords:** bioelectronics, electrophysiology, implants, neuroscience, peripheral nerve, rodent, velocity analysis

## Abstract

Peripheral nerve recordings are a powerful research technique commonly performed using commercially available elastomeric cuff electrodes. However, emerging conformable thin-film technologies which offer advantages in device flexibility, feature miniaturisation, and electrode layout may represent an important alternative tool. In this study, we compare these two device classes as acute recording tools by implanting them side-by-side on rat sciatic nerves under anaesthesia and recording activity evoked by nociceptive muscle stimulation or proprioceptive ankle extension. We find that conventional elastomeric cuffs and conformable thin-film cuffs exhibit no statistically significant signal-to-noise ratio differences under this experimental regime, either under monopolar or referenced recording paradigms. When performing signal velocity analysis, conformable thin-film cuffs show a tendency to toward more frequently classifying recorded sensory activity as afferent, compared to conventional cuffs. Our findings support that conformable thin-film cuffs are competitive research tools for acute peripheral nerve recording.

## 1 Introduction

Peripheral nerve recordings are a useful tool in neuroscience and bioelectronic medicine research. Traditionally, these recordings have been enabled by cuff electrodes consisting of metallic contacts embedded within pre-shaped silicone or similar elastomeric insulating bodies. Such conventional elastomeric cuffs have served as valuable tools for recording neural activity in animal models under anaesthesia, allowing researchers to interrogate peripheral circuits and study fundamental physiological processes. Their applications extend from experimental studies of afferent/sensory and efferent/motor signalling to translational work investigating disease-associated activity in peripheral nerves (Chew et al., 2013; Sabetian et al., 2021; Silverman et al., 2018).

Conformable bioelectronics represent an emerging class of neural interface technology. Photolithographic thin-film fabrication techniques make it possible to produce flexible devices with micrometre-scale thicknesses and feature sizes below 10 μm. These properties offer important advantages for the development of therapeutic medical devices. Their flexibility can improve mechanical compatibility with neural tissue, while their small microelectrodes can enable high-density and spatially resolved recording. These features have made conformable thin-film devices increasingly prominent in central nervous system interfaces (Lee et al., 2025; Liu et al., 2024; Middya et al., 2022; Oldroyd et al., 2024; Woodington et al., 2024). In peripheral nerves, conformable thin-film cuff electrodes have shown reduced foreign body response compared with thicker constructs and have demonstrated reliable recording performance over weeks of implantation (Carnicer-Lombarte et al., 2021), likely driven by their ability to conform closely to the tissue surface.

Despite their therapeutic relevance and good long-term biocompatibility, it remains unclear how the performance of conformable thin-film cuff electrodes compares with that of established conventional elastomeric cuff electrodes under acute recording conditions. The two technologies have generally been developed with somewhat different priorities. Conventional elastomeric cuff electrodes are widely used as robust, accessible research tools for intraoperative experimental recording, whereas conformable thin-film cuff electrodes are being developed as enabling platforms for long-term, high-resolution, or therapy-oriented interfacing. Whether conformable thin-film cuffs could also be used as research tools for acute peripheral nerve recordings, achieving similar performance to conventional cuffs, remains unclear. A direct comparison between the two device classes under matched physiological conditions would therefore be an important step to understand their relative practical advantages as research tools.

In this work, we compare the recording performance of conventional elastomeric cuff electrodes and conformable thin-film cuff electrodes as peripheral nerve recording tools. We implant both classes of device side-by-side on the sciatic nerve of anaesthetised rats, enabling direct within-preparation comparison, and compare recordings obtained during two physiologically relevant stimulation paradigms: nociception and proprioceptive stimulation. We compare the relative performance of the devices in both monopolar recording and referenced recording configurations, and we evaluate their ability to identify signal propagation direction. Together, our work provides a comprehensive direct comparison between a commonly used standard and an emerging class of peripheral nerve cuff technologies as tools for neurophysiology research.

## 2 Materials and methods

### 2.1 Conformable thin-film cuff electrodes

Conformable thin-film cuff electrodes were fabricated using protocols similar to those previously reported (Carnicer-Lombarte et al., 2024; Oldroyd et al., 2025). Four-inch wafers (500 μm thick) were rinsed in acetone and isopropanol, then dehydration-baked at 150 °C for 3 min, and were coated with 3 μm parylene (SCS, Labcoter 2, PDS 2010). For metal patterning, wafers were spin-coated with 3.4 μm negative lift-off photoresist AZnLOF 2035 (Microchemicals GmbH, Germany) (500 rpm/5 s at 1000 rpm s^−1^ acceleration, then 3,000 rpm/30 s at 3000 rpm s^−1^ acceleration), soft-baked at 110 °C for 60 s, UV-exposed (MA/BA 6, Suss MicroTec, Germany; 90 mJ cm^−2^), post-exposure baked at 105 °C for 60 s, and developed in AZ726MIF (Microchemicals GmbH, Germany) for 90 s. Patterned wafers were coated with 10 nm titanium and 100 nm gold (E-beam evaporator, Kurt J. Lesker Company); lift-off was performed by overnight acetone submersion followed by acetone and IPA rinses.

PEDOT:PSS was spin-coated (1,500 rpm, 5,000 rpm s^−1^ acceleration, 5 s) and baked for 1 h at 115 °C (heat-treated devices), then soaked three times in DI water overnight to remove excess PSS and low-molecular-weight compounds. A 2.2 μm photoresist etch mask (AZ 5214E, Microchemicals GmbH, Germany) was soft-baked at 110 °C for 60 s, UV-exposed at 150 mJ cm^−2^, and developed in AZ726MIF for 50 s. The PEDOT:PSS layer was then reactive-ion etched (CF4/O2 at 5/50 sccm, 60 mTorr, 150 W; Plasma Pro80 RIE, Oxford Instruments) for 2 min 45 s (GOPS-crosslinked devices) or 1 min 20 s (heat-treated devices). The remaining resist was removed with acetone and isopropanol rinses.

Wafers were then coated with 3 μm parylene. An outline etch mask was patterned with AZ10XT (Microchemicals GmbH, Germany; spin-coated at 500 rpm/5 s at 300 rpm s^−1^ acceleration, then 3,000 rpm/30 s at 2000 rpm s^−1^ acceleration), soft-baked at 110 °C for 120 s, UV-exposed at 600 mJ cm^−2^, and developed in 3:1 DI water:AZ400K (Microchemicals GmbH, Germany) for 2 min 30 s. Wafers were then etched for 10 min 30 s, and remaining resist removed with acetone and isopropanol. The same process was used to pattern AZ10XT for the insulation etch, using the same etch recipe. Etch mask residues were removed with acetone and IPA rinses.

The conformable thin-film cuffs were designed to wrap around the nerve in a spiral configuration. Each device included larger electrodes positioned toward either side of the cuff (120 μm × 800 μm, 0.5 mm distance between them) and smaller electrodes (100 μm diameter, 175 μm inter-electrode distance) located in the central portion of the device. This layout, as described previously (Kasavetov et al., 2026), enables comparison between larger conformable electrodes, closer in scale to conventional cuff contacts, and smaller microelectrodes enabled by photolithographic fabrication.

As highlighted above, thin-film cuff fabrication requires the use of equipment and knowhow which may be difficult to access to laboratories that do not specialise in the design and fabrication of flexible bioelectronic implants. However, the higher spatial resolution, outline design flexibility, channel number, and conductive and insulating material properties available through this technology makes thin-film bioelectronics an attractive alternative to off-the-shelf elastomeric cuffs. As the field of implantable bioelectronics transitions to these newer materials and techniques, it is likely that thin-film cuffs will similarly become available off-the-shelf, carrying many of these same benefits.

### 2.2 Conventional elastomeric cuff electrodes

Conventional elastomeric cuff electrodes are commonly used as research tools in studies for the acquisition of physiological signals from nerves under anaesthetic conditions (Chew et al., 2013; Sabetian et al., 2021; Silverman et al., 2018), being sold by a variety of companies (CorTec GmbH, Microprobes for Life Science, Ardiem Medical, World Precision Instruments, among others). The elastomeric cuff electrodes used in our study were purchased from CorTec GmbH and used as representative examples of established commercial peripheral nerve cuff technology. These devices are composed of a silicone cuff body with a pre-formed defined architecture, supporting metal foil electrodes, making them a good representation of this class of technology. Two types of platinum–iridium (PtIr) cuff electrodes were used. One was a two-contact micro sling cuff with a nominal internal diameter of 0.3 mm, 2 mm of length, with a proximal-distal interelectrode distance of 1 mm. During implantation, this device was kept open to accommodate the larger rat sciatic nerve. The second was a three-contact micro tunnel cuff with a nominal internal diameter of 1.2 mm, 6 mm of length, with a proximal-distal interelectrode distance (for the electrodes at either end of the cuff) of 3.6 mm. For this tunnel cuff, only the two outermost electrodes were used for the analyses described here. Two-contact micro sling cuffs were used in five extension and four nociception recording sessions, while three-contact micro tunnel cuffs were used in two extension and one nociception recording session.

### 2.3 Implantation and electrophysiological recording

All work was performed in accordance with the UK Animals (Scientific Procedures) Act of 1986 and with approval from the University of Cambridge Animal Welfare and Ethical Review Body. Animal procedures were performed under a project licence (PP5478947) by A. Güemes (personal licence I10076024), issued by the UK Home Office. Female Sprague Dawley rats (Charles River UK) were anaesthetised using isoflurane delivered in medical oxygen. Anaesthesia was induced at 5% v/v, maintained at 2.5% during surgical implantation, and reduced to 0.5–1.5% during electrophysiological recording. Medical oxygen was delivered at 1 L/min. Animal body temperature was maintained using a homeothermic blanket during surgical preparation. The blanket was turned off briefly due to the high electrical noise produced by it during electrophysiological recordings, but animal body temperature continued to be monitored to ensure it remained within normal physiological range.

The sciatic nerve was accessed through a dorsal incision above the femur. Approximately 1 cm of nerve was dissected free from surrounding tissue. A conventional elastomeric cuff and a conformable thin-film cuff were then implanted on the same sciatic nerve, approximately 5 mm apart. The proximal-distal order of the two cuff types was alternated across animals and balanced across extension and nociception experimental paradigms. Both the right and left sciatic nerves were used where appropriate, with each implanted nerve being treated as an independent experimental replicate. The experimental replicate throughout this study therefore consisted of individual nerve preparations (i.e., device recordings from one nerve), with the exception of electrode impedance characterisation in Section 3.1 in which individual electrodes within cuffs are the experimental replicates. While bilateral nerves from the same animal are not perfectly independent, this paired within-nerve design accounted for experimental preparation-to-preparation variability while reducing animal use.

Once implanted, the cuffs were connected to an Intan RHS electrophysiology acquisition system. The cuffs were connected to two separate ports for simultaneous recording. Two stainless steel wires, one per headstage, were connected to the respective GND/reference inputs and placed subcutaneously beneath the skin. Signals were acquired with filtering between 1 Hz and 7.5 kHz and sampled at 20 kS/s.

Recording sessions consisted of either a nociceptive or extension physiological stimulation paradigm. For nociceptive stimulation, a hypodermic needle was used to pierce the tibialis anterior muscle. Stimulation sessions alternated 30 s periods of needle stimulation with 30 s periods without stimulation, typically repeated five times. For extension stimulation, the ankle of the implanted leg was extended by gripping the foot, alternating 10 s periods of extension with 10 s periods of flexion, typically repeated ten times. All physiological stimulation sessions were preceded by a baseline recording lasting 2–5 min, during which no stimulus was provided.

### 2.4 Monopolar recording data analysis

Collected electrophysiological data was analysed in MATLAB. Recordings were imported and digitally filtered to remove mains noise. Signals were further filtered into either a low-frequency band between 10 and 200 Hz or a high-frequency band between 200 and 2000 Hz using a fourth-order Butterworth filter. Any data exceeding the baseline recorded value by more than 150-fold was deemed an artefact and removed. This was done by calculating the standard deviation of the entire trace and, whenever data with an absolute value larger than 150 times this value was detected, this together with data 1.5 ms before and after was removed and replaced by a dummy sinusoid wave signal of the same standard deviation as the average of the recording. The dummy signal had a frequency of 1,400 Hz (high-frequency filtered data) or 95 Hz (low-frequency). This artefact replacement was used to prevent any rare high-amplitude events such as motion artefacts from disproportionately influencing RMS calculations. Despite this precaution, no data used in this study contained fragments that had undergone this artefact replacement.

From these processed datasets, root mean square (RMS) amplitude was calculated using a rolling average with a 1 s window and 0.1 s step size, yielding low-frequency and high-frequency RMS datasets. Firing rate was calculated using a similar rolling-window approach over the high frequency dataset. Spikes were detected using a threshold of five times the baseline standard deviation, using a 0.5 ms refractory period between spike detection events on threshold crossing.

For each recording, periods corresponding to baseline and stimulation were identified. The baseline period was typically a 2–3 min window within the early portion of each recording before any physiological stimulation. The stimulation period consisted of the two or three cleanest nociceptive or extension stimuli. These stimuli were selected by a blinded experimenter on the basis the presence of well-defined features such as clear onset of signal, lack of motion artefacts, and stable signal amplitude. The remaining 65 out of 95 stimuli were not used for analysis. Average RMS values were calculated over the baseline and stimulation periods, yielding baseline and stimulation amplitude values. Signal-to-noise ratio (SNR) was calculated as the ratio between stimulation amplitude and baseline amplitude. Firing rate was calculated as the average over the same selected stimulation window.

### 2.5 Referenced recordings

For referenced recordings, acquired data from each electrode was digitally referenced before filtering or further processing. For conventional elastomeric cuff electrodes, the recording from each electrode (at either end of the cuff) was subtracted from its paired electrode to digitally form a bipolar recording. For the larger electrodes in conformable thin-film cuffs, each electrode was referenced to the average signal from electrodes positioned at the opposite end of the cuff, forming a bipolar recording along the nerve axis. For the smaller electrodes in conformable thin-film cuffs, each electrode was referenced to the average signal of all other small electrodes in the cuff, implementing a common-average referencing paradigm.

The referenced recordings were then processed using the same filtering, RMS, SNR, and firing-rate analysis procedures as the monopolar recordings.

### 2.6 Propagation direction analysis

Velocity and directionality analysis were performed by calculating cross-correlations between unreferenced recordings from proximal and distal electrodes of either conventional elastomeric cuff electrodes or conformable thin-film cuff electrodes (Klus et al., 2025). From these cross-correlations, a single value was calculated to represent the degree to which the recorded activity was predominantly afferent or efferent, in the form of an index.

Correlations corresponding to signal velocities slower than 0.5 m/s were excluded from this index calculation. Velocities above 60 m/s were similarly excluded. While the used combination of sampling rate (20 kS/s) and interelectrode spacing may limit delay detection for fast conducting signals, we have previously shown that this technique performs well under these regimes (Klus et al., 2025). All remaining afferent correlations were pooled into one value (*A*), and all remaining efferent correlations were pooled into another (*E*). The afferent-efferent index was then calculated so that the index ranged from −1, representing fully afferent activity, to +1, representing fully efferent activity, using the following formula:

$$\frac{E-A}{|E|+|A|}$$

### 2.7 Statistical analysis

All statistical analyses were performed in MATLAB (R2025b). Unless otherwise stated, data are reported as mean ± standard deviation. Recording performance (SNR and firing rate) was compared across the three electrode groups (PtIr, PEDOT_L, and PEDOT_S) using one-way repeated-measures ANOVA, with implantation (i.e., the individual nerve preparation) treated as the repeated-measures factor, reflecting that all three electrode types were recorded simultaneously from the same nerve. This approach was applied separately to monopolar and referenced recordings, and to both nociceptive and proprioceptive (ankle extension) stimulation paradigms.

To assess the relative contribution of device type versus experimental/surgical variability to recording performance, a two-way ANOVA was performed on high-frequency SNR values, with device type and surgical preparation of the nerve as factors. The proportion of total sum of squares attributable to each factor was used to estimate its relative contribution to overall variability.

Differences in the afferent–efferent propagation index between conventional elastomeric and conformable thin-film cuff electrodes were assessed using paired Student’s *t*-test.

## 3 Results

### 3.1 Cuff device characteristics

**Figure 1:**
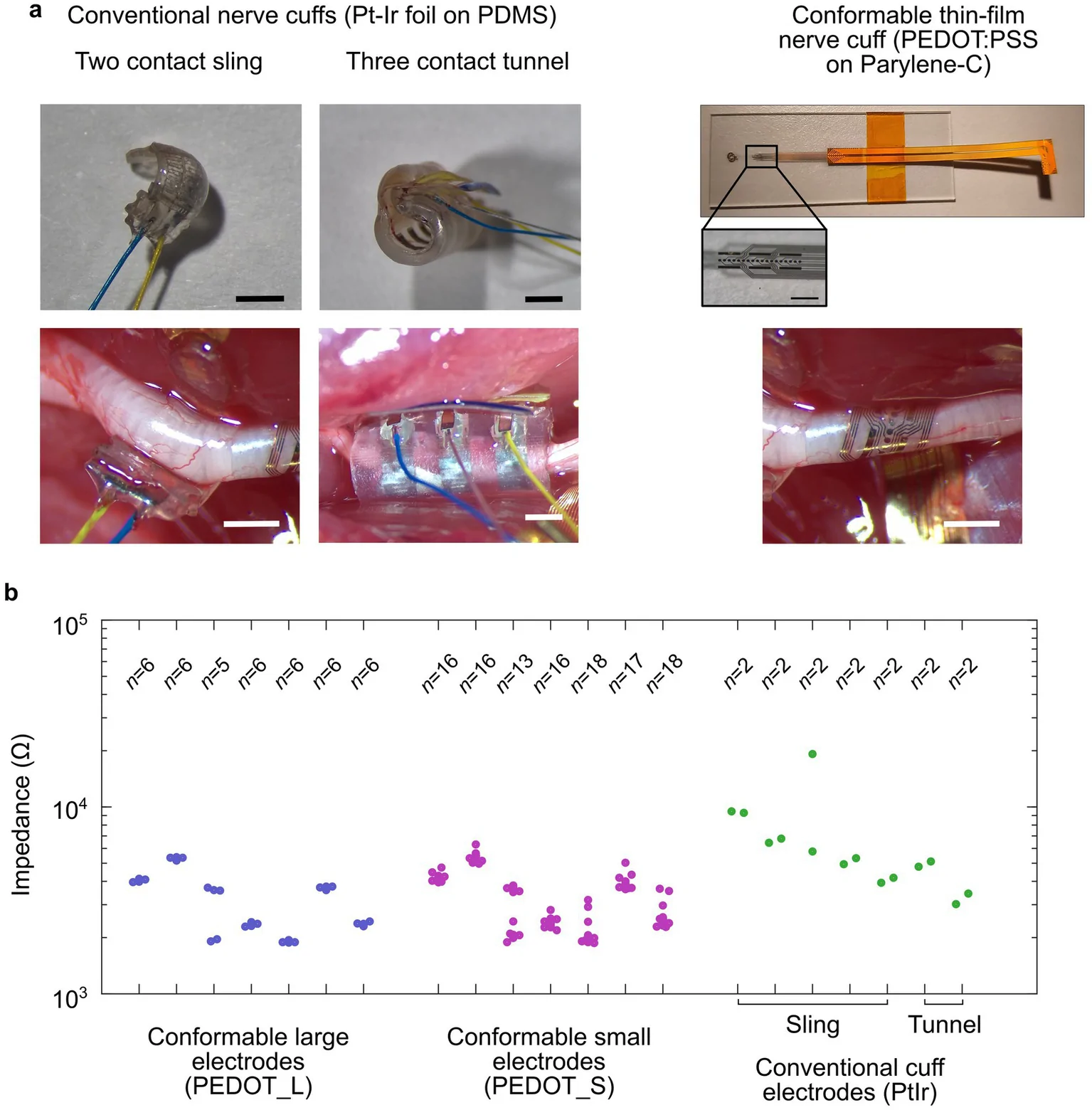
Characteristics of peripheral nerve cuffs. **(a)** Photographs of conventional nerve cuff (two-and three-electrode PDMS and platinum-iridium electrode sling and tunnel) and conformable thin-film cuff (parylene-C and PEDOT:PSS spiral cuff) used in study, before (top) and after (bottom) implantation around the sciatic nerve of a rat. **(b)** Plots of electrode impedances for devices after implantation around nerves. *N* refers to electrode number. Scale bars: 1 mm.

We sought to compare two classes of peripheral nerve cuff electrodes. The first class comprised conventional elastomeric cuff electrodes, represented here by commercial platinum–iridium cuff electrodes (Figure 1a). These included two-contact sling cuffs and three-contact tunnel cuffs. The second class comprised custom conformable thin-film cuff electrodes based on a parylene-C body and PEDOT:PSS electrode sites (Figure 1a). Conventional elastomeric cuffs were implanted either as sling devices drawn close to the nerve or as pre-shaped tunnel cuffs. These devices rely on their cuff geometry to position the electrode contacts around the nerve and generally require appropriate matching between cuff dimensions and nerve size to achieve stable electrode-nerve contact. In contrast, conformable thin-film cuffs were implanted by wrapping the flexible device around the nerve in a spiral configuration, allowing the device body to conform to the nerve surface (Figure 1a).

The conventional elastomeric cuff electrodes contained two electrodes of either 0.5 by 0.5 mm in the sling configuration or 0.6 by 2.4 mm in the tunnel configuration. The conformable thin-film cuff electrodes contained two sets of larger electrodes of 0.12 mm by 0.8 mm positioned at either end of the cuff, as well as a column of smaller electrodes of 0.1 mm diameter located in the middle of the device. Although the device classes differed substantially in dimensions, materials, and implantation mechanics, post-implantation impedance values were broadly similar. Impedance at 1 kHz was 7.53 ± 4.12 kΩ, *n* = 41 electrodes, for conventional elastomeric sling cuffs, 4.09 ± 1.02 kΩ, *n* = 14 electrodes, for conventional elastomeric tunnel cuffs, 3.23 ± 1.16 kΩ, *n* = 114 electrodes, for larger conformable thin-film electrodes, and 3.32 ± 1.17 kΩ for smaller conformable thin-film electrodes (Figure 1b). Throughout this article we refer to conformable thin-film large electrodes as “PEDOT_L”, conformable thin-film small electrodes as “PEDOT_S”, and conventional elastomeric cuffs as “PtIr”.

### 3.2 Recording performance

**Figure 2:**
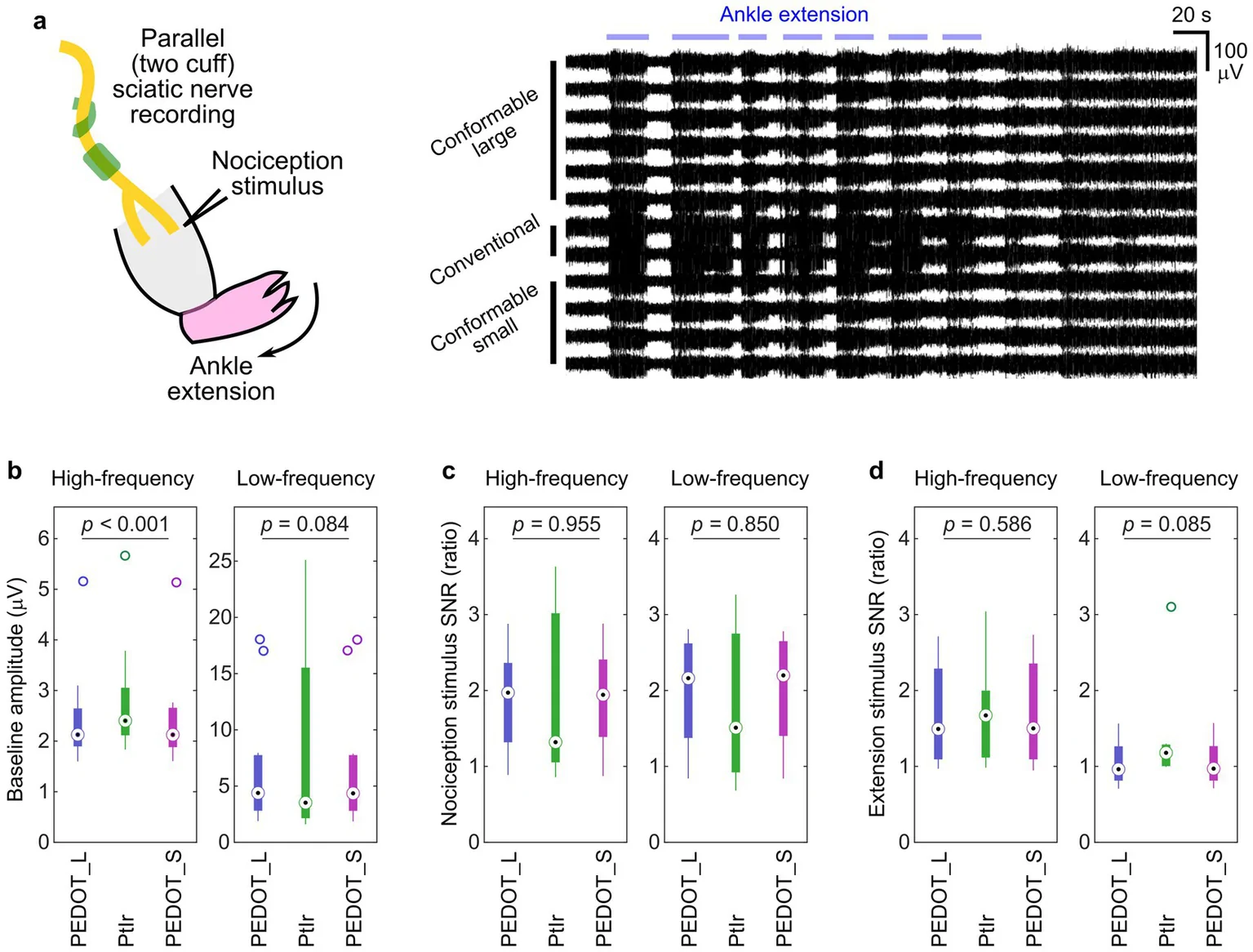
Comparison of nerve cuff recording performance. **(a)** Diagram of experimental design and representative recording. **(b)** Plots of baseline noise amplitude for low- (10 to 200 Hz) and high-frequency (200 to 2000 Hz) bands across nerve electrodes. **(c,d)** Plots of SNR across nerve electrodes under muscle nociceptive **(c)** and ankle extension **(d)** stimuli. Plots consist of boxplots with black dot representing the median value. Statistical test *p* values in plots calculated by one-way repeated measures ANOVA.

We next compared the recording performance of recording electrodes from conventional elastomeric cuffs with the comparable, large electrodes in the conformable thin-film cuffs. We additionally compared performance with smaller electrodes present in the conformable thin-film cuffs as a separate analysis group. Although these smaller electrodes are only achievable through microfabrication-based approaches, they may offer unique advantages for certain research applications, such as high spatial resolution, and therefore represent an important comparison with larger electrode designs. To compare device performance under matched conditions, one conventional elastomeric cuff and one conformable thin-film cuff were implanted on the same sciatic nerve in anaesthetised rats. Recordings were then obtained during physiological stimulation. Two stimulation paradigms were used: nociceptive stimulation, produced by needle insertion into the tibialis anterior muscle, and proprioceptive stimulation, produced by ankle extension (Figure 2a). These two experimental paradigms produce activation of two different nerve fibre populations with distinct firing characteristics (numerous, low amplitude, and slow conducting C fibres carrying nociception; and sparser, higher amplitude, and faster conducting Aα fibres carrying proprioception), thereby allowing for the testing of a range of different recording conditions relevant to nerve electrophysiology.

After implantation, the larger and smaller conformable thin-film electrodes showed similar baseline noise amplitudes. Conventional elastomeric cuff electrodes showed a higher baseline amplitude (Figure 2b, high- and low-frequency average baseline of PEDOT_L: 2.44 and 6.58 mV, PtIr: 2.78 and 8.36 mV, PEDOT_S: 2.40 and 6.54 mV, *n* = 12 devices for each condition – 5 from nociception stimulation experiments, 7 from extension stimulation experiments). This higher baseline amplitude was offset by higher evoked signal amplitudes during both nociceptive and extension stimulation. As a result, SNRs were broadly similar across the three electrode groups during nociceptive stimulation in both low- and high-frequency bands (Figure 2c). A similar pattern was observed during extension stimulation, where conventional elastomeric cuffs again produced larger evoked amplitudes but non-statistically significantly different SNRs relative to conformable thin-film electrodes (Figure 2d).

Although average SNRs were similar across device classes, recording amplitude and SNR varied substantially between implantations. Importantly, this variability often tracked across device types implanted in the same animal and nerve. Preparations in which one cuff type produced high SNRs often also showed high SNRs in the other cuff type, and the same was true for lower-performing preparations. Two-way ANOVA results carried out on high-frequency SNR recordings indicated that most of the result variability corresponded to the animal and surgical setup, with a lower proportion corresponding to the device types for both nociceptive stimulation (Supplementary Figure 1; surgery: 89.29%, device type: 0.12%) and extension stimulation (Supplementary Figure 2; surgery: 96.65%, device type: 0.03%). This suggests that a substantial proportion of the variability was associated with experimental factors such as anaesthetic depth, surgical preparation, or stimulus delivery, rather than intrinsic differences between device classes.

Firing-rate estimates were also calculated across electrode types and stimulation conditions. Firing rates were broadly steady across the three electrode groups during both nociceptive stimulation and extension stimulation (Supplementary Figure 3). Overall, this analysis indicated similar acute recording performance across device categories.

### 3.3 Referenced recording performance

**Figure 3:**
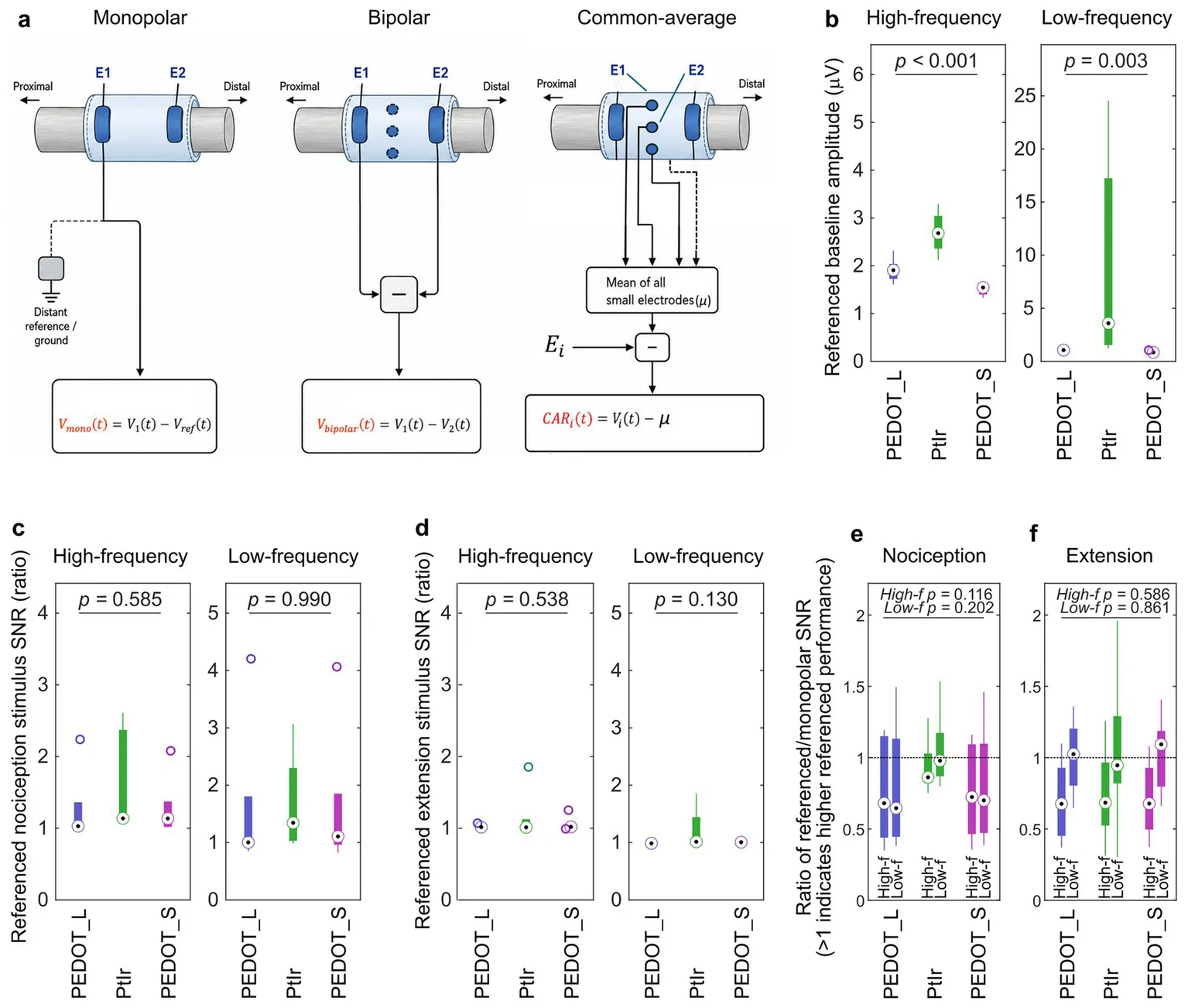
Comparison of nerve cuff recording performance under bipolar and common average referencing regimes. **(a)** Summary of referencing regimes used across device classes and electrodes. **(b)** Plots of baseline noise amplitude for low- (10 to 200 Hz) and high-frequency (200 to 2000 Hz) bands across nerve electrodes following referencing. **(c,d)** Plots of SNR across nerve electrodes under muscle nociceptive **(c)** and ankle extension **(d)** stimuli following referencing. **(e,f)** Plots of the ratios of SNR after referencing compared to before referencing across nerve electrodes under muscle nociceptive **(e)** and ankle extension **(f)** stimuli. High-f and Low-f refer to high- and low-frequency bands, respectively. Plots consist of boxplots with black dot representing the median value. Statistical test *p* values in plots calculated by one-way repeated measures ANOVA. For plots in **(e,f)**, separate ANOVA tests are carried out on high-frequency and low-frequency results.

Having observed broadly similar performance across cuff types in monopolar recordings, we next investigated whether this similarity was also present under common referencing regimes. Electrode referencing is frequently used to reduce common-mode noise and improve SNR in nerve recordings. For conventional elastomeric cuff electrodes and larger conformable thin-film electrodes, we implemented digital bipolar referencing by subtracting recordings from electrodes positioned at opposite ends of the cuff. This configuration removes components common to electrodes aligned along the nerve axis and is a common approach in cuff recordings (Carnicer-Lombarte et al., 2024). For smaller conformable thin-film electrodes, which were arranged as a single circumferential column rather than as distal-proximal electrode pairs, we implemented common-average referencing. This approach uses the average signal across multiple electrodes to subtract components common to the array (Ludwig et al., 2009) (Figure 3a).

Referencing substantially reduced baseline amplitude for conformable thin-film large and small cuff electrodes but not conventional cuff ones, relative to monopolar recordings. This effect was observed in both low-frequency and high-frequency bands (Figure 3b, high- and low-frequency average baseline of PEDOT_L: 1.91 and 1.09 mV, PtIr: 2.69 and 8.46 mV, PEDOT_S: 1.51 and 0.87 mV). However, referencing also reduced the amplitude of stimulation-evoked signals. Consequently, the reduction in baseline amplitude did not translate into a consistent improvement in SNR. This was observed during nociceptive stimulation (Figure 3c) and during extension stimulation (Figure 3d). A similar pattern was seen in firing rate analysis, with most devices showing a decreased performance compared to monopolar recordings (Supplementary Figure 4).

To further quantify the effect of referencing, we calculated the ratio between the SNR obtained from referenced recordings and the corresponding SNR obtained from monopolar recordings. Across all three electrode groups, referencing tended to decrease performance relative to monopolar recording. This decrease appeared more pronounced in larger and smaller conformable thin-film electrodes, although the difference was not statistically significant. This pattern was observed for both nociceptive stimulation (Figure 3e) and extension stimulation (Figure 3f). These results indicate that, in this experimental preparation, referencing did not result in better performance from one over the other device class, and overall decreased recording SNR.

### 3.4 Signal directionality detection performance

By placing electrodes along the axis of the nerve, cuff electrodes can detect small delays in signal arrival between recording sites. These delays can be used to infer the direction and velocity of propagating neural activity. To investigate whether this signal velocity analysis performance was different across device classes, we carried out a signal directionality analysis for both conventional elastomeric cuff electrodes and larger conformable thin-film electrodes using a cross-correlation-based approach.

**Figure 4:**
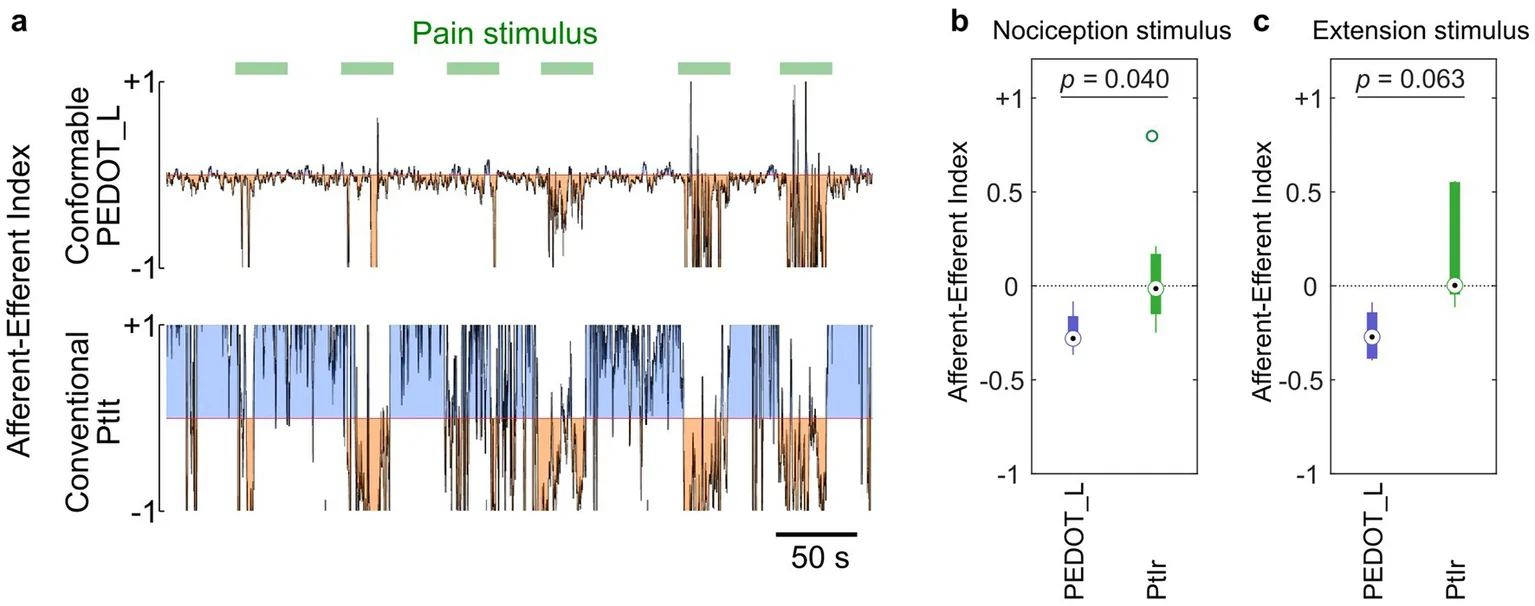
Comparison of velocity analysis across nerve cuffs. **(a)** Example of recorded traces and calculated afferent-efferent index for a pair of conventional nerve cuff (top) and conformable thin-film cuff electrodes (bottom). **(b,c)** Plots of average afferent-efferent index across nerve electrodes under muscle nociceptive **(b)** and ankle extension **(c)** stimuli. Statistical test *p* values in plots calculated by paired Student’s *t*-test. Plots consist of scatter plots with mean value indicated by a red line.

The analysis classified recorded activity as either predominantly afferent or efferent and summarised this classification using an afferent-efferent index, with +1 representing fully efferent signal, and −1 fully afferent signal. Because nociceptive stimulation and ankle extension are expected to evoke sensory activity, both paradigms were expected to produce predominantly afferent signals in the recorded sciatic nerve signals. During both stimulation paradigms, conformable thin-film electrodes showed a stronger trend toward afferent signal detection than conventional elastomeric cuff electrodes (Figure 4a).

While this experimental setup has some limitations, such as lacking a ground truth of the evoked signal directionality, and potential directionality contamination from non-nerve sources such as movement, EMG, reflex activity, or spontaneous motor activity despite the anaesthetised nature of the experiments – this result suggests that conformable thin-film cuff electrodes may provide improved acquisition for propagation-direction analysis. However, both device classes showed strong afferent signal acquisition during periods of physiological stimulation. The main difference between device classes appeared to arise during baseline recordings, where conventional elastomeric cuff electrodes more often showed activity classified as efferent (Figures 4b,c, nociception PEDOT_L: −0.26 ± 0.14, nociception PtIr: 0.20 ± 0.33, extension PEDOT_L: −0.25 ± 0.10, extension PtIr: 0.08 ± 0.35, mean ± standard deviation). This baseline-associated activity may have contributed to a reduced afferent bias in conventional elastomeric cuffs. Since baseline amplitudes were also higher in these devices (Figure 2b), this activity may reflect non-stimulus-related signals with apparent directionality. Mean, standard deviation, and *n* values for these results and those of Figures 2, 3, as well as distribution of animals and nerves across experimental paradigms are presented in Supplementary Figures 5–6. While this study used individual nerve preparation as experimental replicates to reduce animal number, overall statistical results are similar to those obtained when treating individual animals as experimental replicates (Supplementary Figure 7).

## 4 Discussion

The largest source of variability in this study appeared to be associated with the experimental preparation rather than with device class. Factors such as anaesthetic depth, surgical exposure, nerve condition, stimulus delivery, and recording setup likely accounted for much of the variation observed across implantations. This interpretation is supported by the observation that signal-to-noise ratio often tracked across device classes within the same preparation. In other words, when one device performed well in a given animal or nerve, the other device often did too. Surgical implantation of the cuffs may also have contributed to variability, as differences between device classes did not follow a consistent pattern across implantations. In some preparations, conventional elastomeric cuff electrodes performed better; in others, conformable thin-film cuff electrodes performed better. Indeed, in our two-way ANOVA of high-frequency SNR, device type accounted for only 0.03–0.12% of total variance, compared with 89–97% attributable to surgical/animal preparation, a difference of two to three orders of magnitude. This effect-size disparity suggests that, under the acute whole-nerve recording conditions tested here, any true difference between device classes is small relative to the variability inherent to this experimental preparation. Furthermore, we interpret these results as an indication that experimental preparation is a key point of optimization to achieve good quality nerve electrophysiology recordings, overshadowing differences between different cuff implant technologies.

We identify several limitations in our study. First, recordings were obtained under acute anaesthetised conditions in the rat sciatic nerve, and the relative performance of the two cuff technologies may differ in other peripheral nerves with different anatomical dimensions (e.g., nerve diameter) and physiological properties (e.g., fibre thickness and myelination proportion). Second, while both cuff types were compared within the same nerve preparation to minimise experimental variability, some dependence may exist between bilateral nerve recordings obtained from the same animal, such as inter-animal sensitivity to the anaesthetic. Third, the number of replicates used in this study was comparatively small. This poses challenges when testing for assumptions such as normality relating to statistical tests. Finally, the conventional elastomeric cuff group included both sling and tunnel architectures, and the study was not powered to evaluate these designs independently.

Overall, our results indicate that there is no evidence of a significant difference in recording performance between conventional elastomeric cuff electrodes and conformable thin-film cuff electrodes for standard whole-nerve recording. Under these conditions, both device classes were capable of detecting physiologically evoked activity during nociceptive stimulation and ankle extension in our experiments. Therefore, in studies where experimenters need to choose between either device class for acute whole-nerve recording, it is reasonable to carry out this selection based on practical considerations such as technology availability, surgical experience with either technology, and the compatibility of the various device options with the target nerve. Conventional elastomeric cuff electrodes have the advantage of being commercially available and are offered in a range of sizes and configurations. However, because they are typically manufactured with defined geometries, their performance often depends on matching the device to the nerve. Conformable thin-film cuff electrodes are less widely available commercially and require specialised fabrication, being currently often limited to academic laboratory fabrication environments, but their conformable nature allows them to wrap around nerves rather than relying on a fixed pre-shaped geometry. This may allow a single device design to accommodate a broader range of nerve sizes.

A further advantage of conformable thin-film cuff electrodes is their compatibility with high-density, small-dimension electrode layouts. In this study, smaller conformable thin-film electrodes showed recording performance that was overall not statistically different to that of larger conformable electrodes and conventional elastomeric cuff electrodes in standard whole-nerve recordings. This is important because it suggests that miniaturisation does not substantially compromise recording performance in this experimental context. At the same time, smaller electrodes and denser arrays may offer important advantages in applications where spatially resolved recording is required. Prior work has shown that conformable thin-film cuff electrodes can record activity with sub-nerve resolution (Carnicer-Lombarte et al., 2024). Such capabilities may be particularly useful in nerves with spatially organised fascicular structure or in applications where different regions of the nerve are expected to carry distinct functional information.

Conformable thin-film cuff electrodes did show a potential advantage in propagation-direction analysis. Signal velocity and direction propagation analyses are an emerging class of techniques available in nerve electrophysiology. By leveraging the small time differences of recorded signals across nerve cuff electrodes, these techniques allow for the classification of nerve signals to provide insight into their physiological role based on the classified direction (e.g., sensory, motor) and velocity (e.g., slow nociceptive, fast proprioceptive) (Metcalfe et al., 2014; Carnicer-Lombarte et al., 2024; Klus et al., 2025). These devices identified a stronger trend toward afferent activity during both nociceptive and extension stimulation, consistent with the expected sensory nature of the stimuli. While this observation reached statistical significance only in the nociceptive paradigm, it was consistent across both paradigms and should be interpreted as a preliminary finding requiring confirmation in larger studies. We speculate that this may be related to lower baseline activity in conformable thin-film cuffs and better isolation of physiologically relevant neural signals. In conventional elastomeric cuff electrodes, non-afferent activity appeared to be more prominent during baseline periods rather than during stimulation. Since baseline amplitudes were also higher in these devices, this activity may have included non-neural signals or non-stimulus-related neural activity with apparent directionality. Such baseline components could reduce the specificity of directionality analyses, even when stimulation-evoked afferent activity is still clearly detected, suggesting a specific advantage of conformable cuffs in this regard. In summary, this study finds that conformable thin-film cuff electrodes exhibit similar recording performance to conventional elastomeric cuff electrodes under controlled acute experimental conditions. Conventional elastomeric cuff electrodes remain practical and accessible tools for standard peripheral nerve recordings, particularly when a suitable cuff geometry is commercially available for the target nerve. Conformable thin-film cuff electrodes, while currently less accessible, offer important advantages in electrode layout flexibility, compatibility with smaller electrodes, and reduced dependence on specific pre-shaped cuff geometries. These properties make them a competitive alternative as research tools, especially for applications requiring adaptable device geometry, high-density recording, or more advanced analyses such as signal propagation direction detection.

## Data Availability

The datasets presented in this study can be found in online repositories. The names of the repository/repositories and accession number(s) can be found at: https://doi.org/10.6084/m9.figshare.32894972.
