# Supplementary material for: Conformable thin-film cuff electrodes as tools for peripheral nerve recording: a comparative study
Source: Front Neurosci. 2026 Sep 9;20:1930438. doi: 10.3389/fnins.2026.1930438 (PMC13597831; doi:10.3389/fnins.2026.1930438)
Supplement: Supplementary file 1 [file Data_Sheet_1.DOCX]

Supplementary Material

|  | Sum of Squares | Degrees of Freedom | MS | F | p value |
| --- | --- | --- | --- | --- | --- |
| Device types | 0.0127 | 2 | 0.0063 | 0.046 | 0.9553 |
| Surgery | 9.2755 | 4 | 2.3189 | 16.8661 | 0.0006 |
| Error | 1.0999 | 8 | 0.1375 |  |  |
| Total | 10.388 | 14 |  |  |  |

**Supplementary Figure 1.** Two-way ANOVA results for high-frequency (200 to 2000 Hz) monopolar recording SNR under nociceptive stimulation.

|  | Sum of Squares | Degrees of Freedom | MS | F | p value |
| --- | --- | --- | --- | --- | --- |
| Device types | 0.0029 | 2 | 0.0014 | 0.0598 | 0.9422 |
| Surgery | 8.429 | 6 | 1.4048 | 58.1966 | 0.0000 |
| Error | 0.2897 | 12 | 0.0241 |  |  |
| Total | 8.7216 | 20 |  |  |  |

**Supplementary Figure 2.** Two-way ANOVA results for high-frequency (200 to 2000 Hz) monopolar recording SNR under proprioceptive ankle extension stimulation.

**Supplementary Figure 3.** Plots of firing rate across nerve electrodes under muscle nociceptive (a) and ankle extension (b) stimuli. Plots consist of boxplots with black dot representing the median value. Statistical test *p* values in plots calculated by one-way repeated measures ANOVA.

**Supplementary Figure 4.** Plots of firing rate across nerve electrodes under muscle nociceptive (a) and ankle extension (b) stimuli following referencing. Plots consist of boxplots with black dot representing the median value. Statistical test *p* values in plots calculated by one-way repeated measures ANOVA.

| **Experiment** | **PEDOT_L** | | **PtIr** | | **PEDOT_S** | |  | |
| --- | --- | --- | --- | --- | --- | --- | --- | --- |
|  | **Mean** | **Standard deviation** | **Mean** | **Standard deviation** | **Mean** | **Standard deviation** | ***n*** | ***Animals*** |
| **Monopolar, high-frequency, baseline amplitude** | 2.44 | 0.96 | 2.78 | 1.06 | 2.40 | 0.94 | 12 | 7 |
| **Monopolar, low-frequency, baseline amplitude** | 6.56 | 5.49 | 8.36 | 8.45 | 6.54 | 5.50 | 12 | 7 |
| **Monopolar, high-frequency, nociception SNR** | 1.88 | 0.75 | 1.95 | 1.21 | 1.90 | 0.75 | 5 | 3 |
| **Monopolar, low-frequency, nociception SNR** | 1.98 | 0.80 | 1.81 | 1.09 | 2.00 | 0.80 | 5 | 3 |
| **Monopolar, high-frequency, extension SNR** | 1.69 | 0.68 | 1.72 | 0.71 | 1.71 | 0.70 | 7 | 4 |
| **Monopolar, low-frequency, extension SNR** | 1.05 | 0.30 | 1.41 | 0.76 | 1.06 | 0.31 | 7 | 4 |
|  |  |  |  |  |  |  |  |  |
| **Referenced, high-frequency, baseline amplitude** | 1.91 | 0.22 | 2.69 | 0.40 | 1.51 | 0.11 | 12 | 7 |
| **Referenced, low-frequency, baseline amplitude** | 1.09 | 0.10 | 8.46 | 9.30 | 0.87 | 0.07 | 12 | 7 |
| **Referenced, high-frequency, nociception SNR** | 1.27 | 0.54 | 1.64 | 0.75 | 1.28 | 0.45 | 5 | 3 |
| **Referenced, low-frequency, nociception SNR** | 1.61 | 1.45 | 1.70 | 0.87 | 1.62 | 1.37 | 5 | 3 |
| **Referenced, high-frequency, extension SNR** | 1.02 | 0.02 | 1.14 | 0.33 | 1.05 | 0.09 | 7 | 4 |
| **Referenced, low-frequency, extension SNR** | 0.99 | 0.02 | 1.22 | 0.35 | 1.01 | 0.04 | 7 | 4 |
|  |  |  |  |  |  |  |  |  |
| **Ratio referenced/monopolar, nociception** | 0.77 | 0.38 | 0.94 | 0.20 | 0.76 | 0.35 | 5 | 3 |
| **Ratio referenced/monopolar, extension** | 0.69 | 0.28 | 0.76 | 0.32 | 0.70 | 0.26 | 7 | 4 |
|  |  |  |  |  |  |  |  |  |
| **Afferent-Efferent index, nociception** | -0.26 | 0.14 | 0.20 | 0.33 | - | - | 5 | 3 |
| **Afferent-Efferent index, extension** | -0.25 | 0.10 | 0.08 | 0.35 | - | - | 7 | 4 |

**Supplementary Figure 5**. Summary of mean, standard deviation, and replicate number *n* (representing individual nerve preparations) for experiments in Figures 2-4. Note that the PEDOT_S was not used in Afferent-Efferent index experiments.

| **Experimental paradigm** |  |  |  |  |  |  |  |
| --- | --- | --- | --- | --- | --- | --- | --- |
| **Nociception** | Rat1_LN | Rat1_RN | Rat2_LN | Rat2_RN | Rat3_RN | Rat4_LN | Rat4_RN |
| **Extension** | Rat1_LN | Rat1_RN | Rat2_LN | Rat2_RN | Rat3_RN |  |  |

**Supplementary Figure 6**. Summary of rat and nerve preparation breakdown across experimental paradigms. Four rats were used across the two experimental paradigms (Rat1, Rat2, Rat3, Rat4). Their left nerve (LN) and/or right nerve (RN) were used as experimental replicates for each paradigm as per the table..

|  | **Nerve preparation as experimental replicate** | **Animal as experimental replicate** |
| --- | --- | --- |
|  | **Repeated measures ANOVA p value** | |
| **Monopolar, high-frequency, baseline amplitude** | 0.000378 | 0.000248 |
| **Monopolar, low-frequency, baseline amplitude** | 0.083636 | 0.087073 |
| **Monopolar, high-frequency, nociception SNR** | 0.955282 | 0.942089 |
| **Monopolar, low-frequency, nociception SNR** | 0.850118 | 0.671246 |
| **Monopolar, nociception firing rate** | 0.845424 | 0.980153 |
| **Monopolar, high-frequency, extension SNR** | 0.585941 | 0.766251 |
| **Monopolar, low-frequency, extension SNR** | 0.08507 | 0.069574 |
| **Monopolar, extension firing rate** | 0.574364 | 0.941959 |
|  |  |  |
| **Referenced, high-frequency, baseline amplitude** | 0 | 0 |
| **Referenced, low-frequency, baseline amplitude** | 0.002811 | 0.014637 |
| **Referenced, high-frequency, nociception SNR** | 0.584576 | 0.962837 |
| **Referenced, low-frequency, nociception SNR** | 0.990819 | 0.895642 |
| **Referenced, nociception firing rate** | 0.091799 | 0.370223 |
| **Referenced, high-frequency, extension SNR** | 0.538386 | 0.557641 |
| **Referenced, low-frequency, extension SNR** | 0.129784 | 0.189758 |
| **Referenced, extension firing rate** | 0.383496 | 0.413458 |
|  |  |  |
| **Ratio referenced/monopolar, high-frequency, nociception** | 0.115561 | 0.546224 |
| **Ratio referenced/monopolar, low-frequency, nociception** | 0.202487 | 0.806433 |
| **Ratio referenced/monopolar, high-frequency, extension** | 0.585941 | 0.789853 |
| **Ratio referenced/monopolar, low-frequency, extension** | 0.860943 | 0.670555 |
|  |  |  |
|  | **Student’s paired t-test p value** | |
| **Afferent-Efferent index, nociception** | 0.040311 | 0.081373 |
| **Afferent-Efferent index, extension** | 0.062606 | 0.129153 |

**Supplementary Figure 7**. P values for repeated measures ANOVA tests when treating nerve preparations as experimental replicates (i.e. as shown in Figures 2 to 4), compared to when treating individual animals as experimental replicates.
